# ﻿Karyotypic description and comparison of Litoria (L.) paraewingi (Watson et al., 1971), *L.ewingii* (Duméril et Bibron, 1841) and *L.jervisiensis* (Duméril et Bibron, 1841) (Amphibia, Anura)

**DOI:** 10.3897/compcytogen.18.129133

**Published:** 2024-08-20

**Authors:** Richard Mollard, Michael Mahony, Matt West

**Affiliations:** 1 Melbourne Veterinary School, Faculty of Science, The University of Melbourne, Parkville, 3052, Australia; 2 School of Environmental and Life Sciences, University of Newcastle, Callaghan, New South Wales, 2308, Australia; 3 School of Biosciences, Faculty of Science, The University of Melbourne, Parkville, 3052, Australia

**Keywords:** Cell culture, cryopreservation, karyotype, Plains brown tree frog

## Abstract

The karyotype of Litoria (L.) paraewingi (Watson et al., 1971) (Big River State Forest, Victoria) is described here for the first time. It is prepared following tissue culture of toe clipping macerates, cryopreservation, reculture and conventional 4’,6-diamidino-2-phenylindole (DAPI) staining. The *L.paraewingi* karyotype is then compared to similarly processed IUCN (International Union for the Conservation of Nature) least concern members *L.ewingii* (Duméril et Bibron, 1841) (southern Victoria) and *L.jervisiensis* (Duméril et Bibron, 1841) (Myall Lakes National Park, New South Wales), all members of the same *L.ewingii* complex/group. The *L.paraewingi* diploid number is 2n = 26, the same as for the other two species. *Litoriaparaewingi* chromosomes 1, 2, 6 and 7 are submetacentric, chromosomes 3 and 5 are subtelocentric and the remainder are metacentric. No secondary constriction or putative nucleolus organiser region (NOR) was readily identifiable following conventional DAPI staining in any scored *L.paraewingi* metaphase spread. Conversely, a putative NOR was readily identifiable on the long arm of chromosome 1 in all examined metaphase spreads for the other two species. The karyotypes of *L.ewingii* and *L.jervisiensis* here further differ from *L.paraewingi* with chromosome 1 being metacentric and chromosomes 8 and 10 being submetacentric for both former species. The *L.jervisiensis* karyotype differs from those of *L.ewingii* and *L.paraewingi* by DAPI staining with: (i) apparent relative length inversion of subtelocentric chromosome 3 and metacentric chromosome 4 and (ii) chromosome 6 being metacentric rather than submetacentric. All three species have a highly conserved chromosome morphology with respect to chromosomes 2, 5, 7, 9, 11, 12 and 13. The greatest gross morphological difference karyotypically is observed between *L.paraewingi* and *L.jervisiensis*. These karyotype data support the previous phylogenetic separation of these three species based upon genetic compatibility and behavioural, biochemical and molecular genetic analyses.

## ﻿Introduction

The current large scale existential threat to over 40% of amphibian species globally is well documented, making amphibians the most endangered vertebrate taxonomic class (Luedtke et al. 2023). Habitat loss facilitating disease spread, pollutant introduction and species invasion means that for many of these species, animal husbandry, assisted reproductive technologies and cryobanking programs, whether alone or in combination, are suggested requirements for their long-term survival (Kouba et al. 2013; Gillespie et al. 2016; Lampert et al. 2022). Cryobanking initiatives for amphibian assisted reproduction technologies, however, are currently restricted to sperm cells, with methods for oocyte or embryo cryopreservation remaining challenging (Clulow and Clulow 2016; Gagarinskiy et al. 2023). Numerous publications describe somatic cell culture of amphibian somatic cells (Fukui et al. 2003; Ferris et al. 2010; Strauß et al. 2011; Mollard 2018; Mollard et al. 2018; Bui-Marinos et al. 2022; Douglas et al. 2023). It is envisioned that cryopreservation of amphibian somatic cells will provide a necessary immediate resource for longer term genetic conservation initiatives including induced pluripotent stem cell technologies for cloning and gamete production (Kouba et al. 2013; Clulow and Clulow 2016; Codner et al. 2016; Oikawa et al. 2022). In respect to mouse ES cells, maintenance of > 50% euploid karyotype is essential for successful cloning outcomes and by proxy gamete production (Kusakabe et al. 2001; Olifent et al. 2002; Bonnet-Garnier et al. 2015). Cryobanking initiatives of somatic cells aimed at longer term conservation outcomes, therefore, must include steps to ensure recovery of karyotypically normal cells.

A generic level classification of taxa within the Australo-Papuan hyloid family Pelodryadidae has remained problematic largely due to the lack of a comprehensively sampled and well resolved phylogeny for these frogs. The family comprises 232 species split roughly half in Australia and half in Melanesia and eastern Indonesia and contributes 28% of anuran species diversity in the region. Molecular phylogenetic analysis indicates Pelodryadidae diverged approximately 50 million to 100 million years ago while the Australian/ New Guinean land mass and Antarctica were separating (Duellman et al. 2016; Feng et al. 2017; Brennan et al. 2023). Three genera, *Litoria* (Tschudi et Agassiz, 1838), *Cyclorana* (Steindachner, 1867) and *Nyctimystes* (Stejneger, 1916) have been used to taxonomically allocate diversity within the Pelodryadidae, but the description of possible new genera remains the subject of debate (Wells and Wellington 1985; Duellman et al. 2016; Frétey and Dubois 2016). The Australian species of this family are found in all major habitats of the continent (Vidal‐García and Keogh 2015). Despite this ecological breadth, morphological and life history variations are recognised to show a strong association with ecological specialisations. As such, previously applied informal sub-familial classification as species groups accommodating this variation remain widely recognised (Tyler and Davies 1978). One of the well characterised species groups is the Litoria (L.) ewingii group, which comprises nine species (Parkin et al. 2024): *L.callicelis* (Parkin et al., 2024), *L.ewingii* (Duméril et Bibron, 1841), *L.jervisiensis* (Duméril et Bibron, 1841), *L.littlejohni* (White et al., 1994), *L.paraewingi* (Watson et al., 1971), *L.revelata* (Ingram et al., 1982), *L.sibilis* (Parkin et al., 2024), *L.watsoni* (Mahony et al., 2020) and *L.verreauxii* (Duméril, 1853). Assignment to this complex is based upon a range of methods, including: genetic compatibility, mating call comparisons, biochemical analyses and most recently, detailed morphological, mitochondrial genetic and small nucleotide polymorphism (SNP) analyses (Mahony et al. 2020; Parkin et al. 2024).

Despite the in depth molecular analysis underpinning critical phylogenetic assignment within this complex, 2n = 26 karyotypes have been described in the literature for only *L.ewingii*, *L.jervisiensis*, *L.littlejohni* and *L.verreauxii* (Woodruff 1972; White et al. 1994; Schmid et al. 2018). Confirmation of diploid number, position of nucleolus organiser regions (NORs), centromeric positions and relative chromosomal length in karyograms of individual representatives of this complex remain wanting.

Here somatic cells from *L.paraewingi*, *L.ewingii* and *L.jervisiensis* were cultured and cryopreserved in liquid nitrogen (LN2) as a resource to safeguard against possible future existential threats. The previously undescribed karyotype of *L.paraewingi* is compared to that of *L.ewingii* and *L.jervisiensis* following recovery from cryopreservation. All three karyotypes show a 2n = 26 karyotype, yet also differ in several key respects. Most notably, the morphologies of chromosomes 1, 8 and 10 are common to *L.ewingii* and *L.jervisiensis* but not to *L.paraewingi*. A secondary restriction and potential NOR are identified on the long arms of chromosome 1 of both *L.ewingii* and *L.jervisiensis*, but not *L.paraewingi*. The obscure *L.paraewingi* secondary restriction perhaps more closely relates to the more obscure NOR of *L.littlejohni* which is located subterminally on the long arm of chromosome 11 and where satellites are not always observed (White et al. 1994). Karyotypes prepared from the cryobanking of cells from these three species reinforce their phylogenetic separation and provide assurance of relevantly cryopreserved cell types for any required future conservation initiative.

## ﻿Methods

### ﻿Ethics

This research was conducted in compliance with the EU Directive 2010/63/EU for animal experiments and according to The Declaration of Helsinki World Medical Association Code of Ethics. Prior to experimentation, all required Australian State governmental and institutional ethics, licenses and permissions were provided (Richard Mollard, Victorian Department of Environment, Land, Water & Planning Permit number 10008085). The *L.ewingii* specimen was collected from southern Victoria by Richard Mollard under an Animal Ethics Committee Notification of Scavenged Animal Tissue, University of Melbourne. The *L.jervisiensis* specimen was collected by Michael Mahony under the New South Wales National Parks Scientific Licence SL00190. The *L.paraewingi* specimen was collected from Big River State Forest, Victoria, Australia by Matthew West under the Victoria Wildlife Research Permit No. 10009587).

### ﻿Tissue culture and cryopreservation

Toe clippings were obtained from deceased and unsexed *L.ewingii* and *L.jervisiensis* and a male *L.paraewingi*. Culture, cryopreservation, thawing and DAPI karyotyping were performed according to previously described methods (Mollard 2018; Mollard et al. 2018; Mollard and Mahony 2023). Chromosomes were arranged in size by descending order, with the largest chromosome designated chromosome 1 (King et al. 1990). Centromeric position and relative lengths were determined using Image J software with the Levan plugin (Levan et al. 1964). Metacentric, submetacentric and subtelocentric chromosomal morphologies were defined as long arm to short arm ratios of 1–1.69, 1.7–2.99 and 3–6.99, respectively (Levan et al. 1964). Four metaphase spreads each from *L.ewingii* and *L.paraewingi* and eight metaphase spreads from *L.jervisiensis* were arranged in descending order of size for putative NOR assignment, centromeric location and relative length measurements to chromosome 1, not including any secondary constrictions. An extra four *L.jervisiensis* metaphase spreads were scored to accurately compare relative lengths of chromosomes 3 and 4. Images were captured at 1000 × under oil immersion with an Olympus BX60 microscope, colour CCD Leica DFC425C camera, EL-6000 Leica light source and Leica LAS-AF and QCapture Pro7 Version 7.0.5 Build 4325 software (QImaging Inc, USA).

**Figure 1.**
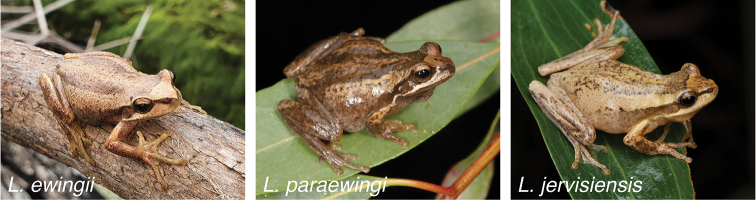
*L.ewingii*; photographed by Matthew West at Merri Creek, Australia, 2020. *L.paraewingi*; photographed by Stephen Mahony at Wangaratta, Victoria, Australia, 2017. *L.jervisiensis*; photographed by Stephen Mahony at Mungo Brush Park Myall Lakes National Park, New South Wales, Australia, 2021.

Cells were processed in culture from toe clippings of *L.ewingii*, *L.paraewingi* and *L.jervisiensis* (representative species images shown in Fig. 1). Following 15, 15 and 3 month LN2 cryopreservation periods, respectively, *L.ewingii*, *L.paraewingi* and *L.jervisiensis* cells were thawed into 24 well plates and passaged for alternate karyotyping and recryopreservation.

## ﻿Results

Of the first 23 *L.ewingii* metaphases spreads scored, 16 (70%) showed a 2n = 26 chromosome count, with the remaining metaphase spreads showing 22 chromosomes (number of spreads = 2), 24 chromosomes (number of spreads = 2) and 25 chromosomes (number of spreads = 3) chromosomes. Of the first 15 *L.paraewingi* metaphase spreads, 13 (87%) showed a 2n = 26 chromosome count with the remaining showing either 23 or 25 chromosomes. Of the first 71 *L.jervisiensis* metaphase spreads scored, 67 (94%) showed a 2n = 26 chromosome count, with the remaining showing either 16, 21, 24 or 25 chromosomes. A higher number of *L.jervisiensis* metaphase spreads were prepared to accurately resolve this species’ unique chromosomal relative length order as outlined below. Reconstruction of the anomalous karyotypes did not reveal obvious aneuploidies such as trisomies or chromosomal pair loss or repeated aneuploidies. Diversion from the 2n = 26 count is most likely technical, therefore, attributable to loss of individual chromosomes during cell dropping and spreading for preparation of DAPI staining and scoring.

**Figure 2.**
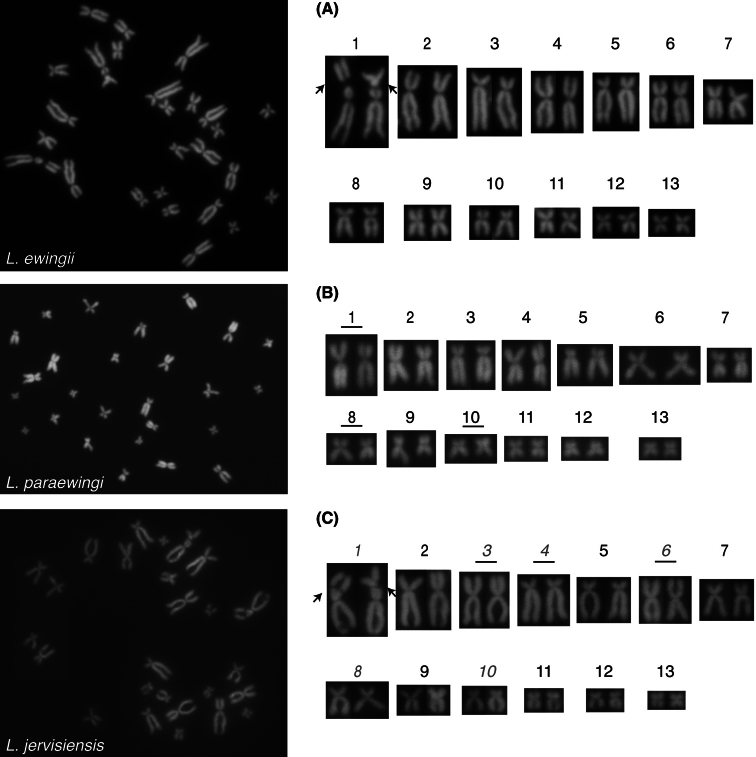
Representative metaphrase spreads of cryopreserved, thawed and cultured cells **A***L.ewingii***B***L.paraewingi***C***L.jervisiensis*. Arrows indicate DAPI negative regions, or presumptive NORs. No DAPI negative regions were apparent in the *L.paraewingi* metaphase spreads. As per Table 1, underlined numbers represent those morphologies differing to *L.ewingii* and italicised (in red) numbers represent *L.jervisiensis* morphologies differing to those of *L.paraewingi*.

**Figure 3.**
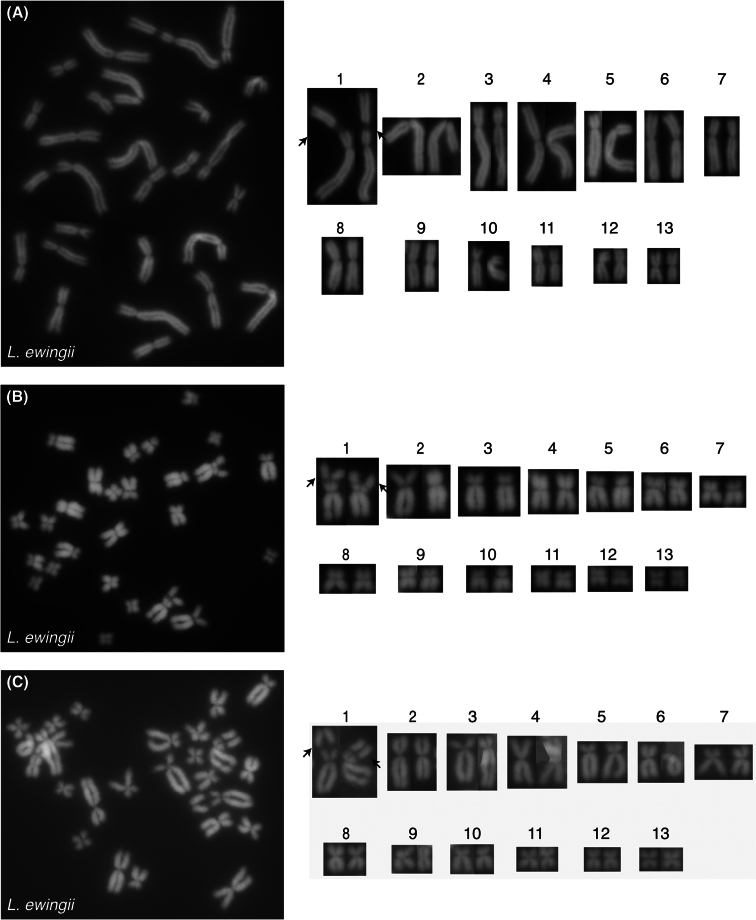
Metaphrase spreads of cryopreserved, thawed and cultured cells from *L.ewingii*. **A–C** three individual metaphase spreads. Arrows indicate DAPI negative regions, or presumptive NORs.

**Figure 4.**
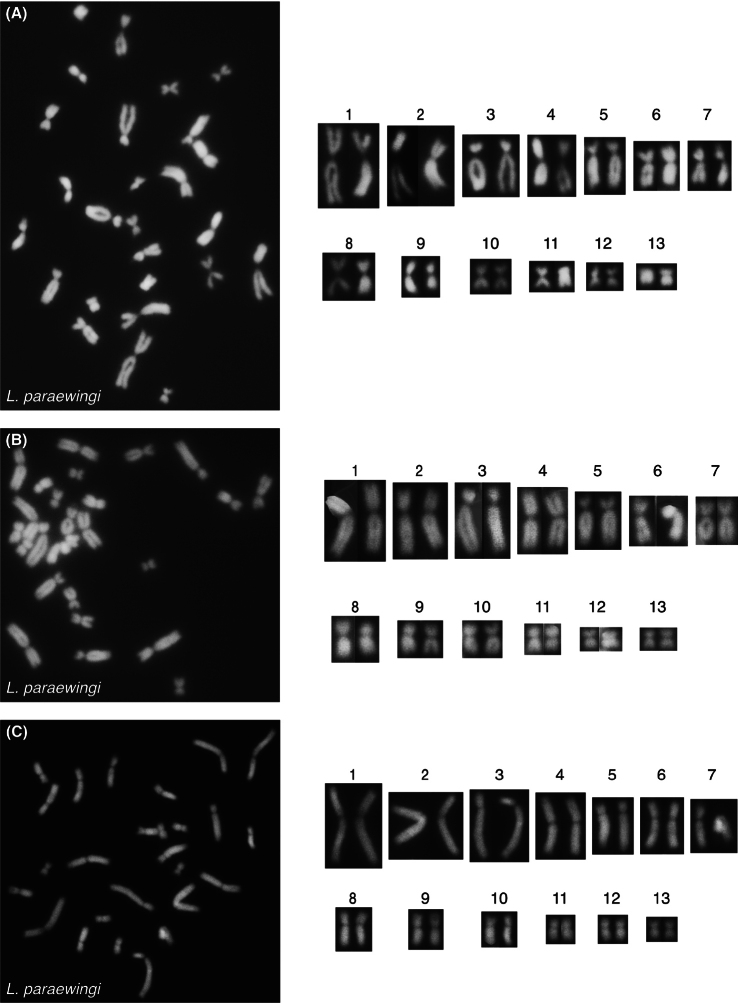
Metaphrase spreads of cryopreserved, thawed and cultured cells from *L.paraewingi*. **A–C** three individual metaphase spreads. No DAPI negative regions, or presumptive NORs, were apparent.

**Figure 5.**
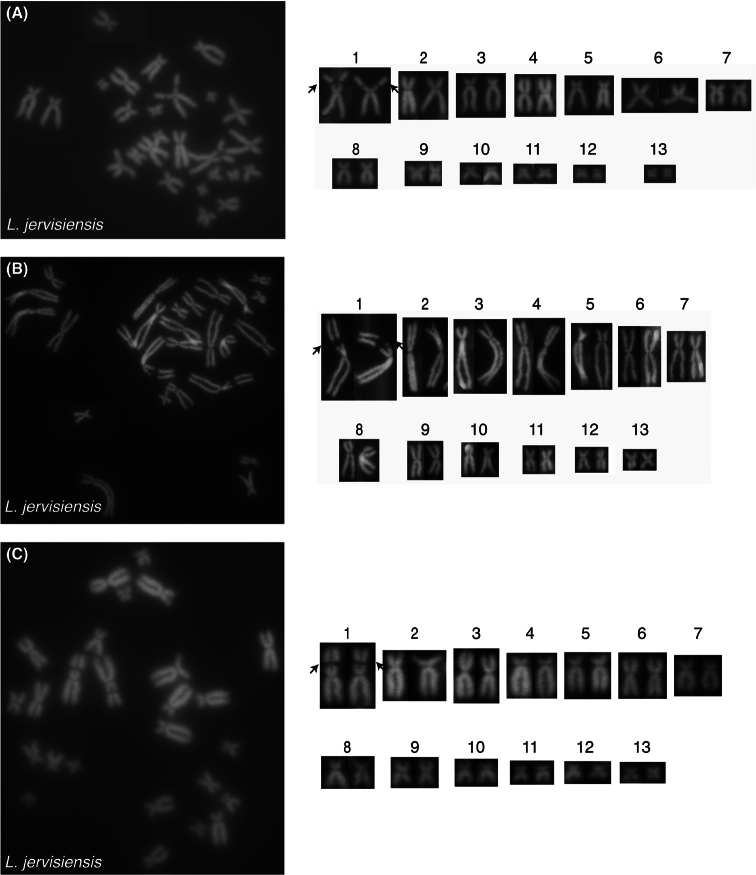
Metaphrase spreads of cryopreserved, thawed and cultured cells from *L.jervisiensis*. **A–C** three individual metaphase spreads. Arrows indicate DAPI negative regions, or presumptive NORs.

For *L.ewingii*, chromosomes 2, 6, 7, 8 and 10 are submetacentric, chromo- somes 3 and 5 are subtelocentric and chromosomes 1, 4, 9, 11, 12, and 13 are metacentric (Table 1, Figs 2, 3A–C). A DAPI negative region was apparent on the long arms of chromosome 1 of all scored *L.ewingii* metaphase spreads and presumed to represent an NOR (Figs 2, 3A–C.) For *L.paraewingi* chromosomes 1, 2, 6 and 7 are metacentric, chromosomes 3 and 5 are subtelocentric and chromosomes 4, 8, 9, 10, 11, 12 and 13 are metacentric (Table 1, Figs 2, 4A–C). No DAPI negative chro- mosomal region was apparent in any of the 15 *L.paraewingi* metaphase spreads (Figs 2, 4A–C). For *L.jervisiensis*, chromosomes 2, 7, 8 and 10 are submetacentric, chromosomes 4 and 5 are subtelocentric and chromosomes 1, 3, 9, 11, 12, and 13 are metacentric (Table 1, Figs 2, 5A–C). A DAPI negative region was apparent on the long arms of chromosome 1 of all scored *L.jervisiensis* metaphase spreads and presumed to represent an NOR (Figs 2, 5A–C).

**Table 1.** Centromeric position (morphology) and relative lengths of chromosomes following DAPI staining of metaphase spreads. Measurements were taken from four *L.ewingii*, four *L.paraewingi* and eight *L.jervisiensis* metaphase spreads. Long arm to short arm ratios (A.R) and relative lengths (R.L.) are provided as average plus or minus standard deviation for all scored metaphase spreads of that species. R.L. is to chromosome 1, designated as length = 1. Chromosomal morphologies (Morph) in cells with light grey shading represent those differing to *L.ewingii*. Italicised chromosomal morphologies represent *L.jervisiensis* morphologies differing to those of *L.paraewingi*.

***Litoriaewingii* Chromosome Number**							
	**1**	**2**	**3**	**4**	**5**	**6**	**7**
**A.R**	1.33 ± 0.12	1.86 ± 0.26	3.38 ± 0.73	1.32 ± 0.10	3.35 ± 0.42	1.86 ± 0.25	1.92 ± 0.36
**Morph**	Metacentric	Submetacentric	Subtelocentric	Metacentric	Subtelocentric	Submetacentric	Submetacentric
**R.L.**	1	0.771	0.7198	0.6833	0.5977	0.5418	0.4185
	**8**	**9**	**10**	**11**	**12**	**13**	
**A.R.**	1.78 ± 0.58	1.59 ± 0.42	2.06 ± 0.50	1.30 ± 0.19	1.33 ± 0.15	1.21 ± 0.16	
**Morph**	Submetacentric	Metacentric	Submetacentric	Metacentric	Metacentric	Metacentric	
**R.L.**	0.4039	0.3533	0.3435	0.2797	0.2785	0.2539	
***Litoriaparaewingi* Chromosome Number**							
	**1**	**2**	**3**	**4**	**5**	**6**	**7**
**A.R.**	1.81 ± 0.26	1.87 ± 0.33	3.78 ± 0.72	1.41 ± 0.30	3.43 ± 0.41	1.97 ± 0.44	1.97 ± 0.38
**Morph**	Submetacentric	Submetacentric	Subtelocentric	Metacentric	Subtelocentric	Submetacentric	Submetacentric
**R.L.**	1	0.8937	0.8609	0.7374	0.6351	0.6192	0.5297
	**8**	**9**	**10**	**11**	**12**	**13**	
**A.R.**	1.52 ± 0.24	1.54 ± 0.24	1.55 ± 0.35	1.46 ± 0.38	1.36 ± 0.20	1.45 ± 0.28	
**Morph**	Metacentric	Metacentric	Metacentric	Metacentric	Metacentric	Metacentric	
**R.L.**	0.4585	0.4108	0.3643	0.2909	0.2532	0.1966	
***Litoriajervisiensis* Chromosome Number**							
	**1**	**2**	**3**	**4**	**5**	**6**	**7**
**A.R.**	1.12 ± 0.09	2.27 ± 0.18	1.41 ± 0.14	3.93 ± 0.48	3.69 ± 0.62	1.36 ± 0.14	2.24 ± 0.29
**Morph**	*Metacentric*	Submetacentric	*Metacentric*	*Subtelocentric*	Subtelocentric	*Metacentric*	Submetacentric
**R.L.**	1	0.7927	0.7116	0.7098	0.6112	0.5978	0.5022
	**8**	**9**	**10**	**11**	**12**	**13**	
**A.R.**	1.92 ± 0.32	1.24 ± 0.16	2.25 ± 0.56	1.53 ± 0.32	1.59 ± 0.36	1.24 ± 0.21	
**Morph**	*Submetacentric*	Metacentric	*Submetacentric*	Metacentric	Metacentric	Metacentric	
**R.L.**	0.4187	0.3439	0.3087	0.2348	0.2067	0.1769	

## ﻿Discussion

Somatic cells from *L.paraewingi*, *L.ewingii* and *L.jervisiensis* were successfully cryobanked in this study with respect to demonstrating recovery of karyotypically normal cells following freeze-thaw cycles. Karyotypes of all three species showed common morphologies for chromosomes 2, 5, 7, 9, 11, 12 and 13, but also unique morphologies. For example, *L.paraewingi* chromosomes 1, 8 and 10 differed morphologically to those of *L.ewingii* and *L.jervisiensis*. The *L.jervisiensis* karyotype differed from those of *L.ewingii* and *L.paraewingi* with respect to an apparent inverted relative length assignment for its metacentric chromosome 3 and subtelocentric chromosome 4. Furthermore, a secondary restriction was discernible on the long arms of chromosome 1 for *L.ewingii* and *L.jervisiensis* but not for *L.paraewingi*. The greatest number of chromosome morphological differences was observed between *L.paraewingi* and *L.jervisiensis*.

*L.paraewingi* is considered a cryptic species due to its high holotypic similarity to *L.ewingii*, with differentiation based upon detailed call analysis, genetic compatibility and molecular taxonomic analysis (Watson et al. 1971; Mahony et al. 2020). Here, by DAPI staining, *L.paraewingi* chromosomes 1, 8 and 10 showed marked divergence from that of *L.ewingii*, providing a further cytological differentiation of these species. The obvious *L.ewingii* and *L.jervisiensis* secondary constriction discernible on the long arms of all chromosome 1 metaphase spreads following DAPI staining is similar to that described previously for *L.verreauxii*, (Schmid et al. 2018). Apparent absence of a DAPI negative region, or secondary restriction, from the karyotype of *L.paraewingi* may be a more similar observation to that reported for *L.littlejohni* (White et al. 1994). For *L.littlejohni* an NOR was discernible sub-terminally on the long arms of chromosome 11 most notably in silver nitrate (Ag-NO_3_) stained chromosomes, with satellites not always observable with conventional aceto-orcein staining. Confirmation of an *L.paraewingi* secondary restriction or NOR location, therefore, may be better resolved in the future by alternative staining techniques such as Ag-NO_3_ staining or 18S or 28S rDNA FISH if also located more terminally without obvious satellites (White et al. 1994; Zaleśna et al. 2017).

## ﻿Conclusion

In conclusion, the karyotypes of *L.paraewingi*, *L.ewingii* and *L.jervisiensis* demonstrate a high level of morphological conservation yet also many unique attributes. These data support the phylogenetic separation of these species based upon previous behavioural, genetic compatibility, biochemical and molecular analyses (Mahony et al. 2020).

## ﻿Competing interests

Richard Mollard has registered a company called Amphicell Pty Ltd (www.amphicell.com). Amphicell Pty Ltd received no funding for this work and privately provided the materials to execute the experimental procedures described in this study.
